# The role of physical activity and diet on bone mineral indices in young men: a cross-sectional study

**DOI:** 10.1186/1550-2783-10-43

**Published:** 2013-09-25

**Authors:** Selma C Liberato, Josefina Bressan, Andrew P Hills

**Affiliations:** 1 Menzies School of Health Research, Charles Darwin University, Darwin, NT, Australia; 2 Departamento de Nutrição e Saúde, Universidade Federal de Viçosa, Viçosa, MG, Brazil; 3 Mater Mother’s Hospital/Mater Research Institute, Griffith Health Institute/Griffith University, Brisbane, QLD, Australia

**Keywords:** Dietary intake, Physical activity, DXA, BMC, BMD, Body composition

## Abstract

**Background:**

Osteoporotic fractures are a significant cause of morbidity and mortality, particularly in developed countries. Increasing peak bone mass in young people may be the most important primary prevention strategy to reduce the risk of osteoporosis. This study aimed to examine the relationship between dietary factors and physical activity on bone mineralization in young men.

**Methods:**

Thirty-five healthy men aged 18–25 y had anthropometric measures, body composition, resting metabolic rate, blood pressure, blood lipids, food intake, physical activity and cardiorespiratory fitness assessed.

**Results:**

Participants who consumed more than 1000 mg/d of calcium were taller and had higher levels of whole body mineral content than participants who consumed less than 1000 mg/d of calcium. Similarly, participants who expended more than 20% of total daily energy engaged in moderate- to vigorous-intensity physical activity had higher cardiorespiratory fitness and higher levels of body mass adjusted bone mineral content than participants who did not meet this level of energy expenditure. There were no differences in blood pressure or blood lipids between participants in calcium or in physical activity energy expenditure categories.

**Conclusions:**

A high intake of dietary calcium and high daily energy expenditure engaged in moderate- to vigorous-intensity physical activity were positively associated with bone mineralization in young men, particularly in the lumbar region.

## Background

Osteoporotic fractures, particularly in the most susceptible areas of the spine and hip [1], are a significant cause of morbidity and mortality in developed countries [2]. Osteoporosis is defined as a reduction in bone mineral density (BMD) 2.5 standard deviation (SD) below the mean for healthy young people at the age of attainment of peak bone mass, in general using a reference population matched for age, sex and race (and expressed as a T-score) [3].

Osteoporotic fractures may have their genesis during the growing years [2] as bone mass and strength are gained during this period [4] with peak bone mass being a major determinant of osteoporosis in later life [5,6]. Therefore, increasing peak bone mass in young people during the time of skeletal maturation may be the ‘best bet’ primary prevention strategy to reduce the likelihood of this disease [6].

While bone and body size have been identified as the main determinants of bone mineral content (BMC) [7], physical activity (PA), nutritional factors, sex hormones and drugs have also been found to play a role in bone mineralization [6-8].

Positive relationships between dairy product intake and total BMC and BMD have been reported in women aged 18–50 y [6,9]. However, it is uncertain which nutrient or combination of nutrients is responsible for changes in bone mass when dairy products are consumed because protein, calcium, phosphorus and vitamin D are known to be associated with bone health [6].

There is limited evidence of an effect of dietary calcium intake on BMC in children [10], young women aged 19–35 y [11] and perimenopausal women aged 45 to 58 y with amenorrhoea for 2–24 months [12]. In adolescents aged 12 to 16 y [8], dietary calcium had no effect on BMC [8].

Physical activity (PA) on the other hand, has been shown to contribute to bone mass in many studies [10,11,13-16]. For example, BMC was found to be higher in the dominant arm of female tennis players [14] and in pre- and early-pubertal children with the highest levels of habitual PA [10] or involvement in a 2-year school-based exercise program [5]. A study with 2384 young men attending the mandatory tests for selection to compulsory military service in Sweden found that history of regular physical was the strongest predictor and could explain 10.1% of the variation in BMD [17].

Type of PA has also been shown to contribute to bone mineralization. Whereas vigorous-intensity PA, including resistance training programs and high-impact exercise has been shown to influence bone mass in some studies [7,15,18-20], others have shown that a minimum intake of calcium seems to be essential for PA to have an impact on bone mass [4,21]. In contrast, strength training 3 d/wk for 12 months had no benefit on bone mineralization in postmenopausal women [21] and there was no association between bone mineralization and level and frequency of sports participation in adolescents aged 12 to 16 y [8].

Calcium and weight-bearing PA have been suggested to have their greatest effect early in life [4,16,22] and with consistently high calcium intake [4,21,23]. The recommended dietary intake (RDA) of calcium for men aged 19–30 y is 1000 mg/d [24] with most young men able to meet the RDA by consuming at least three servings of milk, cheese or yogurt daily. In Australia, the median intake of calcium in men 19–24 y was only 961.5 mg/d [25].

There is limited evidence of an effect of dietary calcium intake on bone mineralization in young men. Studies examining the effects of calcium intake and level of physical activity in free living conditions on bone mineralization are also limited, particularly in young men. In addition, intake of dairy products, which are the main source of calcium [26], may be associated with a dietary fat intake [6] and adversely affect blood lipids [24] or blood pressure. Only one study with girls examining effect of calcium and bone mineralization has investigated the effects of calcium intake on blood lipids.

This study aimed to examine the relationship between dietary factors, physical activity and bone mineralization in young men. Blood lipids were also assessed in the current study.

## Methods

Thirty-five healthy men aged 18–25 y, recruited from the local community in the city of Brisbane, Australia volunteered for the study. Participants were recruited by flyers posted in shopping centers and education centers as well advertisement in local newspapers. Inclusion criteria to participate in the study were age between 18 and 25 years and absence of any chronic disease. Queensland University of Technology Human Research Ethics Committee approved the participant recruitment and data collection procedures. The methods of this cross-sectional study have been previously described in detail [27] and are here described in brief. Anthropometric measures including body weight and height, body composition, and waist and hip circumferences were undertaken. Body mass index (BMI) was calculated as weight (kg) divided by height (m^2^). Body composition, including BMC, BMD and lean body mass, was measured by dual-energy X-ray absorptiometry (DXA) (DPX-Plus; Lunar Corp, Madison, WI).

Resting metabolic rate (RMR) was assessed by continuous open-circuit indirect calorimetry using a Deltatrac II metabolic cart (Datex-Ohmeda Corp., Helsinki, Finland http://www.hospitalnetwork.com/doc/Deltatrac-II-Metabolic-Monitor-0001) in half of the participants. Due to technical problems, the MOXUS O_2_ system (AEI Technologies, Pennsylvania, USA) was used to assess RMR of the remaining participants. In our laboratories we have consistently found measured RMR values are less than 100 kcal lower using the Deltatrac compared to MOXUS system. A similar proportion of lean and overweight participants were assessed using each of the methods and therefore likelihood of measurement bias was reduced.

Sitting blood pressure (BP) was assessed after a 10-min rest using a standard sphygmomanometer. Following an overnight fast of at least 8 h, a blood sample was collected for later total cholesterol (TC), high density lipoprotein cholesterol (HDL-C), low density lipoprotein cholesterol (LDL-C) and triglycerides (TG) determination using reagents from Roche Diagnostics (Indianapolis, IN). The measurement of TC and LDL-C were based on the determination of Δ^4^-cholestenone after enzymatic cleavage of the cholesterol ester by cholesterol esterase, conversion of cholesterol by cholesterol oxidase, and subsequent measurement by Trinder reaction of the hydrogen peroxide formed [28]. A combination of a sugar compound with detergent was used to selectively determine LDL-C in serum [28]. The HDL-C was determined directly in serum using polyethylene glycol-modified enzymes and dextran sulfate [28].

Both food intake and PA were assessed over four days. Food intake was assessed using household estimates in a food record, and entered into the Foodworks (v.3.02) nutrient analysis software (Xris software Pty Ltd. Brisbane, Australia, http://www.xyris.com.au). Protein and fat were expressed as source of energy intake (EI). As PA has been shown to have no effect with calcium intake <1000 mg/d [21], an average daily intake of 1000 mg of calcium was used as the cut-off to divide participants into low- and high-intake of calcium groups.

Physical activity was assessed based on activity records using nine categories of PA intensity (1–9) to account for each 15-min period throughout the day. The four-day PA record scores 1, 2, 3, 4, 5, 6, 7, 8 and 9 correspond to 1, 1.5, 2.3, 2.8, 3.3, 4.8, 5.6, 6 and 7.8 metabolic equivalents (METs), respectively [29]. Using measured RMR, the total daily energy expenditure (TDEE) was calculated for each participant after accounting for each of the 96 15-min periods of a day and multiplying the score by its specific MET value. Physical activity level was calculated by dividing TDEE by RMR. For each participant, 15-min periods were classified into three PA levels, according to the Center for Disease Control and Prevention and the American College of Sports Medicine Position Statement [30]: a) light (TDEE < 3 METs), moderate (3–6 METs) and vigorous (TDEE ≥ 6 METs). The B-PAR scores 1 to 4, 5 to 7 and 8 to 9 correspond to light, moderate and vigorous PA, respectively [29,31]. A median 20% percent of TDEE engaged in moderate- to vigorous-intensity PA served as the cut-off for high *vs.* lower level PA groups.

Cardiorespiratory fitness was measured by a continuous speed, incremental grade running test on a treadmill. Participants were fitted with a Polar Coded Transmitter™ and receiver (Polar Electro, Kempele, Finland), a Hans-Rudolf headset (with two-way breathing valve and pneumotach) and a nose-clip. After a 4-min warm-up at 3.5 mph, 0% grade, speed was increased to a previously determined comfortable speed, which was the same until the end of the test. Thereafter, the treadmill slope was increased by 2% every min, until the participant reached exhaustion. Rating of perceived exertion using the Borg scale was obtained during each stage and participants were encouraged to achieve a rating of 18 or higher as an indicator of maximal effort. Maximal oxygen uptake (VO_2max_) was assessed using a MOXUS Modular O_2_ System (AEI Technologies, Pennsylvania, USA). VO_2max_ was achieved when the difference between the last 2 completed stages determined by the average of the last 30-sec period before the load increased was <1.6 ml/kg.min or when both heart rate ±10 bpm of 220 – age and respiratory exchange ratio >1.15, were achieved. VO_2max_ was defined as the highest observed value averaged across 15 seconds in a completed stage. When the participant did not reach VO_2max_, VO_2_ peak oxygen uptake, the highest observed value of VO_2_, was considered in analysis.

All measurements were undertaken by the same investigator in two sessions of two hours each. In the first session, after receiving a detailed explanation of the study requirements and measurements to be collected, the participants provided written consent to participate in the study, had anthropometric data and blood pressure collected and had 1-hour session with a dietitian about how to record the dietary intake data. The participants were asked to attend the biochemical laboratory at their convenience to have blood sample collected after a fasting period for the lipids measurements. In the second session, the participants discussed in detail the dietary intake data recorded to clarify any doubts and had the body composition, resting metabolic rate and the cardiorespiratory fitness measured. A technician helped with the body composition and cardiorespiratory fitness assessment.

A two factor, between-subjects analysis of variance was performed. The factorial analysis of variance (ANOVA) is an inferential statistical test that allows testing if each of several independent variables has an effect on the dependent variable. It also allows determination of the independence of main effects (i.e., if two more independent variables interact with each other).

Participants in the current study were divided according to their calcium intake (low and high calcium intake refers to less or more than 1000 g/d, respectively) and percentage of TDEE engaged in moderate-to-vigorous PA (low and high PA refers to expending less or more than 20% of TDEE engaged in moderate-to-vigorous PA, respectively) in a 2 × 2 between-subjects, factorial design. If there was no interaction between independent variables (p > 0.05 for all dependent variables) the variables were independently analysed by *T* test.

## Results

Factorial analysis considering calcium as one factor and PA as the other factor was not significant (p > 0.05) for all variables tested. Therefore, the mean for calcium intake as well as for PA were compared by *T* test.

Anthropometric, PA, fitness, dietary and DXA measurements according to calcium intake and energy expended of the participants are shown in Table 1. Participants who consumed more than 1000 mg/d of calcium were taller and energy-adjusted calcium intake, calcium/phosphorus ratio, and lean mass adjustment calcium intake were higher than participants who consumed less than 1000 mg/d of calcium. Participants who expended more than 20% of the TDEE engaged in moderate- to vigorous-intensity PA had higher VO_2_ max than participants who expended less (Table 1).

**Table 1:** **Anthropometric, physical activity (PA), fitness (VO**_**2 **_**max), dietary and DXA measurements of the participants (mean ± SD) according to calcium dietary intake and PA level**

	**Low calcium**	**High calcium**	**P values**^**1**^	**Low PA**	**High PA**	**P values**^**1**^
Anthropometric						
Height (m)	1.73 ± 0.07	1.78 ± 0.06	0.03	1.74 ± 0.06	1.77 ± 0.08	0.18
Body mass (kg)	74.89 ± 14.50	79.83 ± 12.03	0.28	79.39 ± 13.39	76.12 ± 13.45	0.48
Lean mass (kg)	56.42 ± 8.41	61.47 ± 7.72	0.07	57.37 ± 8.30	60.11 ± 8.39	0.34
BMI (kg/m^2^)	24.79 ± 3.99	25.03 ± 3.03	0.84	26.15 ± 3.77	24.09 ± 3.09	0.09
Waist circumference	82.21 ± 9.06	83.06 ± 7.72	0.77	85.32 ± 9.18	80.86 ± 7.31	0.12
Physical activity						
EE doing moderate to vigorous PA (kcal)	744.62 ± 410.72	988.04 ± 412.21	0.09	477.91 ± 179.90	1131.08 ± 324.14	0.09
VO_2_ max (ml of O_2_)	50.84 ± 8.30	53.26 ± 6.41	0.37	47.38 ± 7.94	54.93 ± 5.48	0.01
Dietary						
Calcium (mg)	757.91	1458.57		1008.20 ± 555.12	1191.62 ± 399.24	0.26
Calcium/energy (mg/kcal)	0.32 ± 0.09	0.50 ± 0.12	< 0.001	0.40 ± 0.19	0.42 ± 0.10	0.64
Calcium/phosphorus	0.49 ± 0.12	0.68 ± 0.10	< 0.001	0.57 ± 0.17	0.61 ± 0.13	0.52
Calcium/lean mass (mg/kg)	0.0135 ± 0.0035	0.0241 ± 0.0070	< 0.001	0.0177 ± 0.0099	0.02 ± 0.01	0.48
Protein (%)	16.92 ± 4.74	16.68 ± 2.52	0.85	17.26 ± 5.04	16.49 ± 2.58	0.61
Fat (%)	32.36 ± 5.79	32.17 ± 4.85	0.92	32.86 ± 6.46	31.86 ± 4.38	0.59

*Abbreviations*: BMI, Body mass index; EE, energy expenditure.

^1^ T test.

Table 2 contains mean values of whole body and regional BMC and BMD according to participants’ calcium intake and energy expenditure engaged in moderate- to vigorous-intensity PA. Participants who consumed more than 1000 mg/d of calcium had higher levels of whole body BMC, height-adjusted whole body BMC, BMI-adjusted whole body BMC, trunk BMC, lumbar L1-L4 BMC, BMI-adjusted lumbar L1-L4 BMC, lumbar L2-L4 BMC and BMI-adjusted lumbar L2-L4 BMC than participants who consumed less than 1000 mg/d of calcium. Participants who expended greater energy had higher levels of body mass adjusted whole body BMC, BMI-adjusted whole body BMC, trunk BMC, body mass adjusted lumbar L1-L4 BMC, BMI-adjusted lumbar L1-L4 BMC, body mass adjusted lumbar L2-L4 BMC and BMI-adjusted lumbar L2-L4 BMC than participants who expended less energy (Table 2).

**Table 2:** Mean values ± SD of body composition parameters in young men having low and high intake of calcium and expending low and high percentage of daily energy engaged in moderate- to vigorous intensity physical activity (PA)

	**Low calcium intake**	**High calcium intake**	**P values**^**1**^	**Low PA**	**High PA**	**P values**^**1**^
BMC (g)						
Whole body	3191.26 ± 555.27	3611.15 ± 486.94	0.02	3263.56 ± 473.83	3502.97 ± 596.04	0.21
Whole body/height	1833.41 ± 267.85	2021.94 ± 239.81	0.04	1872.64 ± 242.08	1968.86 ± 282.55	0.30
Whole body/body mass	42.97 ± 4.61	45.44 ± 3.23	0.07	41.41 ± 3.73	46.13 ± 3.18	<0.001
Whole body/BMI	129.67 ± 12.82	144.57 ± 19.10	0.01	125.39 ± 12.25	145.30 ± 16.26	<0.001
Arms	434.18 ± 85.41	470.52 ± 93.25	0.24	436.66 ± 80.28	463.67 ± 96.48	0.39
Legs	1269.27 ± 251.31	1335.26 ± 232.11	0.43	1266.74 ± 224.37	1327.52 ± 252.87	0.47
Trunk	1056.90 ± 204.60	1209.20 ± 229.90	0.05	1043.53 ± 174.67	1196.36 ± 242.72	0.05
L1L4	94.24 ± 19.30	112.81 ± 21.76	0.01	96.24 ± 19.36	108.83 ± 23.26	0.10
L1L4/body mass	1.28 ± 0.28	1.43 ± 0.31	0.16	1.22 ± 0.20	1.45 ± 0.32	0.02
L1L4/BMI	3.88 ± 0.81	4.53 ± 1.00	0.04	3.70 ± 0.63	4.56 ± 0.99	0.01
L2L4	68.34 ± 13.64	80.71 ± 12.07	0.01	72.31 ± 13.80	76.29 ± 14.46	0.42
L2L4/body mass	0.93 ± 0.18	1.03 ± 0.20	0.14	0.92 ± 0.15	1.02 ± 0.22	0.14
L2L4/BMI	2.80 ± 0.48	3.25 ± 0.65	0.03	2.78 ± 0.43	3.20 ± 0.65	0.04
BMD (g/cm^2^)						
Whole body	1.27 ± 0.10	1.30 ± 0.09	0.35	1.27 ± 0.09	1.30 ± 0.10	0.34
Arms	1.01 ± 0.09	1.04 ± 0.10	0.25	1.02 ± 0.09	1.03 ± 0.10	0.65
Legs	1.44 ± 0.12	1.48 ± 0.13	0.36	1.43 ± 0.11	1.48 ± 0.14	0.29
Trunk	1.04 ± 0.11	1.09 ± 0.09	0.14	1.03 ± 0.09	1.08 ± 0.10	0.07
Lumbar L1L4	1.04 ± 0.15	1.06 ± 0.12	0.69	1.05 ± 0.15	1.06 ± 0.12	0.80
Lumbar L2L4	1.15 ± 0.14	1.16 ± 0.16	0.80	1.14 ± 0.16	1.17 ± 0.14	0.49

*Abbreviations*: BMC, body mineral content; BMD, body mineral density; BMI, Body mass index.

^1^ T test.

There were no between-group differences in blood pressure or blood lipids based either on calcium intake level or on energy expenditure engaged in moderate- to vigorous-intensity PA level (Table 3).

**Table 3:** Serum lipids in the young men having low and high calcium intake and expending low and high percentage of daily energy engaged in moderate- to vigorous- intensity physical activity (PA)

	**Low calcium intake**	**High calcium intake**	**P values**^**1**^	**Low PA**	**High PA**	**P values**^**1**^
Diastolic (mmHg)	119.24 ± 10.12	124.56 ± 9.55	0.12	123.29 ± 7.68	121.10 ± 11.46	0.53
Systolic (mmHg)	59.53 ± 7.73	57.50 ± 6.72	0.41	60.36 ± 7.09	57.24 ± 7.16	0.21
TC (mmol/L)	4.46 ± 1.31	4.45 ± 0.54	0.98	4.60 ± 1.30	4.36 ± 0.71	0.48
HDL-C (mmol/L)	1.39 ± 0.28	1.40 ± 0.24	0.92	1.37 ± 0.21	1.41 ± 0.29	0.68
LDL-C (mmol/L)	2.66 ± 1.01	2.66 ± 0.55	0.99	2.77 ± 1.03	2.59 ± 0.61	0.54
Triglycerides (mmol/L)	1.19 ± 1.4	1.01 ± 0.44	0.61	1.39 ± 1.53	0.90 ± 0.36	0.25
TC/HDL-C	3.32 ± 1.10	3.27 ± 0.65	0.87	3.41 ± 0.99	3.22 ± 0.82	0.53
LDL-C/HDL-C	2.00 ± 0.84	1.98 ± 0.59	0.94	2.06 ± 0.77	1.94 ± 0.68	0.60

*Abbreviations*: TC, Total cholesterol, HDL-C, High density cholesterol, LDL-C, Low density cholesterol.

^1^ T test.

## Discussion

High intake of calcium and high energy expended engaged in moderate- to vigorous-intensity PA was associated with high bone mass in the young men participating in the current study. Higher BMC was observed in whole body, trunk and lumbar regions but not in legs or arms of young men who consumed more than 1000 mg/d of calcium compared to those who consumed less than 1000 mg/d of calcium. There was no difference in the absolute values of bone mineralization but when these values were adjusted for body mass or BMI, participants who expended more than 20% of the EE engaged in moderate- to vigorous- intensity PA had higher of levels of whole body BMC, trunk BMC, Lumbar L1-L4 BMC, and Lumbar L2-L4 than participants who expended less.

The association between high bone mass and high intake of calcium is similar to other studies [9-12,23] as is the relationship between calcium intake and lumbar spine but not legs or arms [18,32,33]. Lumbar spine consists of primarily cancellous bone which is more metabolically active [18] and therefore more responsive to dietary intake and, or PA intervention than peripheral cortical bone [5,8,13,18].

Calcium intake had no effect on any of the BMD measurements in the current study, also consistent with other studies [6,8,10,34]. On the other hand, calcium intake was shown to have an effect on BMD in girls. Positive association between calcium intake and bone mass were reported in young women aged 19–35 y [11] and BMD increased from 11 to 17 y in girls with consistently high calcium intake [23]. Bone mineral density does not account effectively for diverse body sizes [10] and BMC has been suggested to be a better indicator of accretion in bone mineralisation than BMD [6].

The finding of the current study that high intake of calcium did not adversely affect blood lipids or blood pressure is also similar to another study [6]. Supplementation with dairy products to at least 1000 mg/d for 12 months in 91 girls aged 15–16 years did not adversely affect blood lipids [6]. High intake of calcium could have been related to high intake of dairy and consequently high intake of fat. However this was not the case in this study. Intake of fat as a percentage of energy was similar in participants who consumed less or more than 1000 mg/d of calcium.

High nutrient density foods such as low-fat dairy foods were the main sources of calcium for participants who consumed more calcium as evidenced by no between-group differences in protein and fat percentage contribution to EI. Further, participants who consumed more than 1000 mg/d of calcium had higher energy adjusted calcium compared to participants who consumed less.

High protein intake has been shown to produce negative calcium balance from increased urinary calcium excretion if phosphorus intake is kept low [6]. Calcium balance does not seem to have been negative in the participants of the current study because intake of protein was within the recommended intake accounting for more than 16% of the energy intake.

A high Ca/P intake ratio in participants who consumed more than 1000 mg/d of calcium compared to participants who consumed less may also have contributed to a higher bone mass. High Ca/P intake ratio has been shown to be positively associated with bone mass [12,35].

Participants of the current study who expended more than 20% of total energy engaged in moderate- to vigorous-intensity PA had higher VO_2_ max than participants who expended less. This finding indicates that data are reliable despite using subjective measurements to assess PA.

A significant positive effect of moderate- to vigorous-intensity PA was observed on whole body BMC normalized to either BMI or body mass. Whole body BMC normalized to either BMI or body mass may be a better measurement of bone mass because BMC is known to be heavily influenced by body weight, body height and body lean mass. Bones are mineralized, in part, due to forces they are habitually exposed to and therefore larger individuals necessarily expose their bones to larger forces, resulting in higher BMC and BMD [18].

The effects of moderate- to vigorous-intensity PA in participants of the current study were evident in the lumbar spine. Similar findings were observed in other studies with young adults [36,37]. A 12 y follow-up study with participants aged 20–29 y at baseline showed that increased PA was associated with increased BMD at the lumbar spine [36]. A study with 12 men and 12 women aged between 18 and 23 years participating in a resistance training applying loads to the hip and spine for 24 weeks, on three nonconsecutive days per week showed that males had an increase in BMD of 7.7% in the lateral spine L2-L4 while the change in women was 1.5% [37]. A study with resistance athletes, runners and cyclists found that muscle contraction makes a significant contribution to the lean bone mass-associated increases in BMD [38]. Continued heavy training leads to continuous reactivating remodelling [15,21] by replacing damaged and degraded bone tissue with new tissue [15] and increases bone mineralization [7,11,14,16,18].

A small sample size was a limitation of the current study. Another limitation is that RMR of half of the participants was assessed using different equipment due to technical problems. However the likelihood of measurement bias is small because a similar proportion of lean and overweight participants was assessed using each of the equipments. Nevertheless, the findings contribute to a better understanding of the bone mineralization of young Australian men, an important group which has been under-represented in previous work.

## Conclusion

High intake of calcium and high energy expended engaged in moderate- to vigorous- intensity PA were positively associated with bone mineralization particularly in lumbar region of young men.

## Competing interests

The authors declare that they have no competing interests.

## Authors’ contributions

SCL defined the design of the study, undertook data collection, data collation, data analysis and manuscript preparation. JB helped with manuscript writing. APH secured support for this study and helped with manuscript writing. All authors read and approved the manuscript.
